# The Psychological Impact of Hypertension During COVID-19 Restrictions: Retrospective Case-Control Study

**DOI:** 10.2196/25610

**Published:** 2021-03-30

**Authors:** Carissa Bonner, Erin Cvejic, Julie Ayre, Jennifer Isautier, Christopher Semsarian, Brooke Nickel, Carys Batcup, Kristen Pickles, Rachael Dodd, Samuel Cornell, Tessa Copp, Kirsten J McCaffery

**Affiliations:** 1 Sydney Health Literacy Lab, School of Public Health Faculty of Medicine and Health The University of Sydney Sydney Australia; 2 Agnes Ginges Centre for Molecular Cardiology Centenary Institute The University of Sydney Sydney Australia; 3 Faculty of Medicine and Health The University of Sydney Sydney Australia; 4 Department of Cardiology Royal Prince Alfred Hospital Sydney Australia

**Keywords:** public health, global health, COVID-19, hypertension, risk, strategy, mental health, behavior, response, anxiety, vaccine, retrospective, perception, prevention, intention

## Abstract

**Background:**

It is unclear how people with hypertension are responding to the COVID-19 pandemic given their increased risk, and whether targeted public health strategies are needed.

**Objective:**

This retrospective case-control study compared people with hypertension to matched healthy controls during the COVID-19 lockdown to determine whether they have higher risk perceptions, anxiety, and vaccination intentions.

**Methods:**

Baseline data from a national survey were collected in April 2020 during the COVID-19 lockdown in Australia. People who reported hypertension with no other chronic conditions were randomly matched to healthy controls of similar age, gender, education, and health literacy level. A subset including participants with hypertension was followed up at 2 months after restrictions were eased. Risk perceptions, anxiety, and vaccination intentions were measured in April and June.

**Results:**

Of the 4362 baseline participants, 466 (10.7%) reported hypertension with no other chronic conditions. A subset of 1369 people were followed up at 2 months, which included 147 (10.7%) participants with hypertension. At baseline, perceived seriousness was high for both hypertension and control groups. The hypertension group reported greater anxiety compared to the controls and were more willing to vaccinate against influenza, but COVID-19 vaccination intentions were similar. At follow-up, these differences were no longer present in the longitudinal subsample. Perceived seriousness and anxiety had decreased, but vaccination intentions for both influenza and COVID-19 remained high across groups (>80%).

**Conclusions:**

Anxiety was above normal levels during the COVID-19 lockdown. It was higher in the hypertension group, which also had higher vaccination intentions. Groups that are more vulnerable to COVID-19 may require targeted mental health screening during periods of greater risk. Despite a decrease in perceived risk and anxiety after 2 months of lockdown restrictions, vaccination intentions remained high, which is encouraging for the future prevention of COVID-19.

## Introduction

Although research on COVID-19 outcomes is constantly evolving, there is consistent evidence that people with cardiovascular disease (CVD) risk factors are more likely to experience severe complications and are more likely to die if they acquire COVID-19 [1]. People with CVD are more likely to have risk factors that may complicate the response to COVID-19, and COVID-19 can itself cause cardiovascular damage [2]. During the early phase of the pandemic, there was prominent media attention about the risk of hypertension in particular, and there were concerns that people with CVD risk factors were not presenting to general practitioners and hospitals for management and new symptoms onset due to the fear of contracting COVID-19 [3,4]. People with CVD risk factors or established CVD can access prescriptions via telehealth in Australia, but this was very new at the time of the study [5]. As well as potential access issues, many people with chronic conditions do not believe they are at increased risk, which may affect their uptake of prevention measures [6]. This may be reinforced by beliefs based on misinformation about the severity of COVID-19, spread as part of antivaccination movements [7].

In addition to concern about increased risk for this population, there has been debate in the medical community about whether common medications used to manage risk for people with CVD, hypertension, and diabetes contribute to worse COVID-19 outcomes [8,9]. At the time of this study, there was insufficient evidence to cease their use, prompting the National Heart Foundation to release a statement confirming this [10]. However, there continues to be research on the role of angiotensin converting enzyme inhibitors and angiotensin II type I receptor blockers, with arguments both for and against the continued use of such medications [11,12] during the COVID-19 pandemic in different population groups.

There has also been debate about the respiratory versus cardiovascular nature of COVID-19. Emerging research suggests that virus complications and their treatment could be regarded as cardiovascular in nature [13,14], which may explain the devastating outcomes experienced by some people who contract the virus. It is unclear what this means for managing people with multiple CVD risk factors associated with worse COVID-19 outcomes (eg, hypertension and diabetes) [8]. Initial concerns promoted in national media included both respiratory conditions, such as asthma [15], and cardiovascular conditions, including hypertension [16], early in the Australian pandemic response.

As a result of this evolving and conflicting research, as well as widespread misinformation, people with hypertension in the community may have received mixed messages in the media about how they should manage both CVD risk and COVID-19 risk during the pandemic. It is unknown whether people with hypertension responded differently to the pandemic and associated restrictions compared to the general population and whether a tailored communication approach is needed to address the needs of this group.

This study investigated whether people with hypertension have higher risk perceptions, anxiety, and prevention intentions during COVID-19 restrictions to inform targeted public health messaging for this group.

## Methods

### Setting

**Figure 1:**
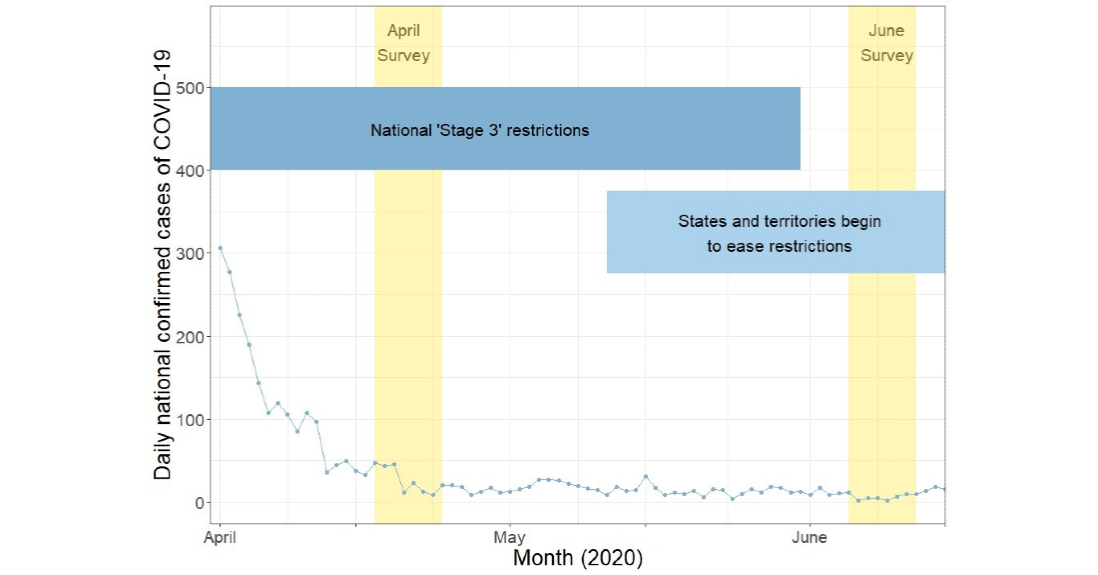
COVID-19 in Australia during the study period.

In Australia, the COVID-19 pandemic has been well controlled compared to many other countries around the world. However, in April 2020, cases and community transmissions had been rising exponentially, and the country was placed under lockdown, including closure of schools and workplaces and restrictions on gatherings and movement. Citizens were required to stay home except for essential purposes (eg, work, essential shopping, exercise). In June 2020, cases were under control and many regulations were eased, although some restrictions remained, such as small gathering sizes, which varied from state to state. A second wave occurred in the state of Victoria shortly after this, requiring new restrictions such as mandatory masks and curfews, but our data were collected prior to this. Thus, a comparison of April and June data presents an opportunity to look at the effect of a short-term lockdown between a time of strong COVID-19 restrictions and good control (Figure 1).

### Data Collection

Data from a national Australian survey were used to conduct retrospective case-control analyses comparing hypertension and control groups. Baseline data were collected from all states and territories in April 2020 during the COVID-19 lockdown, with a subsample followed up in June 2020 when restrictions were eased.

Ethics approval was obtained from the University of Sydney Human Research Ethics Committee (2020/212).

### Measures

The survey measures and full sample results are reported elsewhere [17,18], including the Health Literacy single-item screener [19], Consumer Health Activation Index (CHAI) patient activation measure [20], and State-Trait Anxiety Inventory (STAI) [21]. Participants were asked if they had any of the following conditions: asthma, chronic obstructive pulmonary disease, high blood pressure (hypertension), cancer, heart disease, stroke, diabetes, depression, or anxiety; and whether they take any prescription medication (not specified). The single-item screener provides a brief measure of health literacy, that is, the skills needed to engage in health [19]; and the CHAI provides a measure of patient activation, that is, the extent that a person actively involves themselves in decisions to manage one’s health [20]. Risk perceptions and prevention behaviors (including vaccination intentions) were measured using Likert and categorical scales. Items pertaining to risk perception were based on items developed for an earlier US COVID-19 study [18]. The perceived seriousness of threat from COVID-19 was captured using a 10-point scale (1=”no threat at all to” 10=”very serious public health threat”). The social distancing score reflects perceived importance of social distancing. This outcome is based on 4 items, each answered using a 7-point Likert scale. The items were adapted from existing vaccine attitude instruments to instead reflect on social distancing (“social distancing is important for my family’s health,” “social distancing is important for the health of others in my community,” “when everyone else is socially distancing, I don’t need to,” “I socially distance to protect people with a weaker immune system”). Perceived seriousness was asked generally at baseline; at follow-up, participants were asked about the public health risk from COVID-19 in general, globally, and in Australia specifically, given the divergent pattern of control across countries.

### Matching Procedure

Individuals with hypertension and no other comorbidities (n=466) were retrospectively matched without replacement to healthy controls (with no comorbidities; n=2251) using the *calipmatch* function in Stata (StataCorp) [22]. For each case, potential controls were initially identified based on age (±3 years) and exactly matching on gender, education, and health literacy adequacy (selected given observed differences as a function of these variables in COVID-19–related knowledge, attitudes, awareness, and behaviors in our baseline survey [17]). One matching control is then randomly selected for the case and removed from the list of available controls for subsequent cases. Because the search strategy for controls is greedy (ie, selecting cases for matching in random order and removing controls without replacement for subsequent case matching), some cases may be left unmatched. The initial matching run resulted in 95.7% (446/466) of cases successfully matched to a control. The constraints for matching were iteratively relaxed (eg, allowing age to vary by ±10 years; education level to differ by one category) until all remaining cases were paired to a control. The matching procedure was repeated for the follow-up sample.

### Analysis

Analyses were conducted using Stata/IC v16.1 (StataCorp). Pairwise comparisons of baseline demographic characteristics were undertaken to confirm the appropriateness of the matching procedure of cases to controls, and to identify potential differences in demographic characteristics between those who were invited and returned for follow-up compared to those who were not followed up. Regression models with robust error variances to account for clustering within pairs, and adjusted for matching variables (age, gender, education, and health literacy adequacy), were used to analyze outcome variables. Linear models were used for continuous outcomes (risk perceptions, STAI anxiety, perceived importance of social distancing) to estimate marginal mean differences (MMD). Generalized linear models with a modified Poisson approach [23] were used for the dichotomous outcome “not feeling stressed due to COVID-19,” generating adjusted prevalence ratios (aPR). Ordinal logistic regression models were used for ordered categorical outcomes (frequency of leaving one’s home, vaccination intentions), resulting in adjusted odds ratios (aOR). Separate models were conducted for each time point. All estimates are provided with 95% CI values. A *P* value of .05 was used as the threshold for statistical significance.

### Data Availability

Data are available upon reasonable request subject to ethics approval.

## Results

Of the 4362 baseline participants, 466 (10.7%) reported hypertension with no other chronic conditions. A subset of 1369 participants from the original survey cohort were followed up after 2 months, comprising 147 (10.7%) participants with hypertension only.

Table 1 describes the case versus control samples for all baseline outcomes, and Table 2 shows details of the regression models comparing the two groups at this timepoint. Table 3 provides a description of cases and controls included in the follow-up sample, with Table 4 detailing the outcome of the regression models at follow-up.

**Table 1:** Baseline descriptive statistics and unadjusted outcomes for hypertension cases versus matched healthy controls.

Variable				Group^a^		
				Hypertension (n=466)		Control (n=466)
**Sample description**						
	Age (years), mean (SD)			53.5 (15.5)		52.5 (15.3)
	**Age group, n (%)**					
		18-25 years	26 (6)		34 (7)	
		26-40 years	83 (18)		78 (17)	
		41-55 years	105 (23)		117 (25)	
		56-90 years	252 (54)		237 (51)	
	**Gender, n (%)**					
		Male	220 (47)		220 (47)	
		Female	243 (52)		243 (52)	
		Not specified/other	3 (1)		3 (1)	
	**Education, n (%)**					
		High school or less	115 (25)		112 (24)	
		Certificate I-IV	69 (15)		69 (15)	
		University	282 (61)		285 (61)	
	Adequate health literacy^b^, n (%)			427 (92)		431 (92)
	Takes any prescription medicine, n (%)			359 (77)		195 (42)
	Consumer Health Activation Index (score 0-100 where 100 is more active), mean (SD)			75.83 (14.19)		77.17 (12.77)
**Risk perception**						
	Seriousness of threat (0=low, 10=high), mean (SD)			7.72 (2.25)		7.66 (2.18)
	What percentage of people who get COVID-19 will die as a result? (open), mean (SD)			6.50 (13.49)		5.72 (12.45)
	What percentage of people who get COVID-19 will experience only mild symptoms? (open), mean (SD)			62.88 (26.36)		62.37 (27.12)
**Anxiety**						
	State-Trait Anxiety Inventory (score range 20-80; normal 34-36), mean (SD)			40.62 (14.95)		38.98 (14.38)
	Never (in the past week) felt nervous or stressed because of COVID-19 (categorical), n (%)			113 (24)		115 (25)
**Prevention behaviors**						
	Perceived importance of social distancing (average of 4 items from 1-7, where 7 is most important), mean (SD)			6.48 (0.74)		6.42 (0.82)
	**How often are you leaving home? n (%)**					
		Less than once per week	45 (10)		42 (9)	
		Once per week	53 (11)		53 (11)	
		A few times per week	176 (38)		150 (32)	
		Once per day	154 (33)		176 (38)	
		Multiple times per day	38 (8)		45 (10)	
	**I have or I will get the flu vaccine this year, n (%)**					
		Strongly disagree/disagree	50 (11)		72 (15)	
		Neither agree nor disagree	30 (6)		39 (8)	
		Strongly agree/agree	386 (83)		355 (76)	
	**If a COVID-19 vaccine becomes available, I will get it, n (%)**					
		Strongly disagree/disagree	17 (4)		29 (6)	
		Neither agree nor disagree	45 (10)		42 (9)	
		Strongly agree/agree	404 (87)		395 (85)	

^a^People reporting high blood pressure and no other conditions were matched to healthy controls with no reported cardiovascular or respiratory conditions.

^b^Based on the single-item health literacy screener.

**Table 2:** Multivariable^a^ regression model estimates comparing hypertension cases (n=466) versus matched healthy controls (n=466) at baseline.

Variable		Estimate (95% CI)	*P* value
**Risk perception**			
	Seriousness of threat, MMD^b^	0.05 (–0.23 to 0.34)	.71
	What percentage of people who get COVID-19 will die as a result? MMD	0.75 (–0.87 to 2.37)	.36
	What percentage of people who get COVID-19 will experience only mild symptoms? MMD	0.71 (–2.77 to 4.18)	.69
**Anxiety**			
	State-Trait Anxiety Inventory, MMD	1.90 (0.19 to 3.61)	.03
	Never (in the past week) felt nervous or stressed because of COVID-19, aPR^c^	0.96 (0.77 to 1.19)	.69
**Prevention behaviors**			
	Perceived importance of social distancing, MMD	0.06 (–0.04 to 0.17)	.21
	How often are you leaving home? aOR^d^	0.84 (0.66 to 1.06)	.14
	I have or I will get the flu vaccine this year, aOR	1.52 (1.10 to 2.11)	.01
	If a COVID-19 vaccine becomes available, I will get it, aOR	1.21 (0.84 to 1.73)	.31

^a^All multivariable models controlled for age (in years), gender, health literacy adequacy, and education.

^b^MMD: marginal mean difference (from the linear regression model).

^c^aPR: adjusted prevalence ratio (from the generalized linear model using a modified Poisson approach).

^d^aOR: adjusted odds ratio (from the ordinal logistic regression).

**Table 3:** Follow-up descriptive statistics and unadjusted outcomes for hypertension cases versus matched healthy controls.

Variable					Group^a^			
					Hypertension (n=147)			Control (n=147)
**Sample description^b^**								
	Age (years), mean (SD)			54.8 (14.9)			52.8 (14.2)	
	**Age group, n (%)**							
		18-25 years	7 (5)			8 (5)		
		26-40 years	22 (15)			22 (15)		
		41-55 years	36 (24)			45 (31)		
		56-90 years	82 (56)			72 (49)		
	**Gender, n (%)**							
		Male	61 (41)			61 (41)		
		Female	85 (58)			85 (58)		
		Not specified/other	1 (1)			1 (1)		
	**Education, n (%)**							
		High school or less	26 (18)			18 (12)		
		Certificate I-IV	19 (13)			21 (14)		
		University	102 (69)			108 (73)		
	Adequate health literacy^c^, n (%)			142 (97)			143 (97)	
	Takes any prescription medicine, n (%)			114 (78)			56 (38)	
	Consumer Health Activation Index (score 0-100, where 100 is more active), mean (SD)			75.48 (14.32)			77.10 (12.95)	
**Risk perception**								
	Seriousness of threat in general (0=low to 10=high), mean (SD)			7.51 (2.42)			7.03 (2.58)	
	Seriousness of threat globally (0=low to 10=high), mean (SD)			8.74 (1.76)			8.65 (1.81)	
	Seriousness of threat in Australia (0=low to 10=high), mean (SD)			6.14 (2.38)			5.50 (2.49)	
**Anxiety**								
	State-Trait Anxiety Inventory (score range 20-80; normal 34-36), mean (SD)			36.94 (15.31)			36.49 (13.93)	
	Never (in the past week) felt nervous or stressed because of COVID-19 (categorical), n (%)			58 (39)			64 (44)	
**Prevention behaviors**								
	Perceived importance of social distancing (average of 4 items from 1-7, where 7 is more important), mean (SD)			6.49 (0.78)			6.34 (0.90)	
	**I have or I will get the flu vaccine this year, n (%)**							
		Strongly disagree/disagree	13 (9)			24 (16)		
		Neither agree nor disagree	2 (1)			2 (1)		
		Strongly agree/agree	132 (90)			121 (82)		
	**If a COVID-19 vaccine becomes available, I will get it, n (%)**							
		Strongly disagree/disagree	7 (5)			13 (9)		
		Neither agree nor disagree	9 (6)			10 (7)		
		Strongly agree/agree	131 (89)			124 (84)		

^a^People reporting high blood pressure and no other conditions were matched to healthy controls with no reported cardiovascular or respiratory conditions.

^b^As measured at baseline.

^c^Based on the single-item health literacy screener.

**Table 4:** Multivariable^a^ regression model estimates comparing hypertension cases (n=147) versus matched healthy controls (n=147) at follow-up.

Variable		Estimate (95% CI)	*P* value
**Risk perception**			
	Seriousness of threat in general, MMD^b^	0.50 (–0.08 to 1.08)	.09
	Seriousness of threat globally, MMD	0.07 (–0.31 to 0.46)	.71
	Seriousness of threat in Australia, MMD	0.60 (0.05 to 1.15)	.03
**Anxiety**			
	State-Trait Anxiety Inventory, MMD	0.94 (–2.57 to 4.45)	.60
	Never (in the past week) felt nervous or stressed because of COVID-19, aPR^c^	1.03 (0.94 to 1.12)	.55
**Prevention behaviors**			
	Perceived importance of social distancing, MMD	0.16 (–0.03 to 0.35)	.11
	I have or I will get the flu vaccine this year, aOR^d^	1.90 (0.93 to 3.90)	.08
	If a COVID-19 vaccine becomes available, I will get it, aOR	1.72 (0.82 to 3.58)	.15

^a^All multivariable models controlled for age (in years), gender, health literacy adequacy, and education.

^b^MMD: marginal mean difference (from the linear regression model).

^c^aPR: adjusted prevalence ratio (from the generalized linear model using a modified Poisson approach).

^d^aOR: adjusted odds ratio (from the ordinal logistic regression).

### Description of Sample

To isolate the effects of hypertension, the hypertension sample included 466 people reporting only high blood pressure and no other chronic health conditions. The mean age was 54 years (SD 15.5), and the sample comprised 52% (n=243) female, 47% (n=220) male, and 1% (n=3) unspecified. The majority had a university degree (n=282, 61%) and adequate health literacy (n=427, 92%). The average patient activation score was comparable to other patient populations (mean scaled CHAI 74.9). Most were taking medications (n=359, 77%), with 45% (n=163) obtaining a refill during the lockdown, 5% (n=19) switching to a longer prescription, and only 1 person stopping their medication. As seen in Table 1, the sample descriptive characteristics were comparable between individuals with hypertension and the matched controls. There was no statistical difference across age (*P*=.33), gender (*P*>*.*99), education *(P*=*.*97), or health literacy adequacy (*P*=*.*63) between cases and controls. Cases who were invited and returned for follow-up were of similar age and gender but had higher levels of education (*P*=*.*02) and were more likely to have adequate health literacy (*P*=*.*009) than those who were not followed up.

### Risk Perceptions

At baseline, the perceived seriousness of threat from COVID-19 in the hypertension group was high (mean 7.72, out of 10) but similar to controls (mean 7.66). On average, the hypertension sample believed that 7% of people who get COVID-19 would die as a result and 63% would experience only mild symptoms (asked separately). There were no statistically significant differences between the hypertension group and the matched controls at baseline. At follow-up, those with hypertension perceived a greater threat (mean 6.12) than controls (mean 5.52) when asked about Australia (MMD 0.60, 95% CI 0.05-1.15; *P*=.03) but not in general or globally.

### Anxiety

At baseline, 76% (n=353) of the hypertension group had felt nervous or stressed about COVID-19 in the past week at least some of the time. On average, the mean STAI was 1.90 units higher (95% CI 0.19 to 3.61; *P*=.03, Cohen *d*=0.13) for those with hypertension (mean 40.75) than matched controls (mean 38.85). At follow-up, there was no longer a significant difference between the hypertension (mean 37.02) and control (mean 36.08) groups (MMD 0.94, 95% CI –2.57 to 4.45; *P*=.60, Cohen *d*=0.06).

### Prevention Behaviors

At baseline, the hypertension group had a social distancing score of 6.48 out of 7, indicating strong agreement with the importance of social distancing for ones’ own health and the health of the public; this was similar to the controls (6.42 out of 7). Most people left home a few times a week (n=176, 38%) or once a day (n=154, 33%) during the lockdown. Overall, 83% (n=386) agreed they would get the influenza vaccine, and 87% (n=404) would get the COVID-19 vaccine. Compared to healthy matched controls, the hypertension group was more likely to agree that they would (or have already) received the influenza vaccine this year (aOR 1.52, 95% CI 1.10-2.11; *P*=.01). There were no significant differences in willingness to vaccinate for COVID-19 (if it became available), perceived importance of social distancing, or frequency of leaving one’s home. At follow-up, there was no longer a significant difference between the hypertension and control groups for influenza vaccination intention (aOR 1.90, 95% CI 0.93-3.90; *P*=.08), with intentions remaining high for both influenza and COVID-19 vaccination (>80% for both groups).

## Discussion

### Principal Findings

The main observation of this study was the significant difference in anxiety levels between hypertension only and matched control groups, with all groups reporting higher than “normal” levels. This is consistent with the Australian Bureau of Statistics’ finding that the rate of anxiety in the general population had doubled in April 2020 compared to a survey from 2017-18 [24]. Prioritizing mental health screening for more vulnerable clinical groups with higher anxiety may be warranted when local community transmission rates are high.

Overall, there were few differences between people with hypertension and healthy matched controls. No significant differences were found for COVID-19 risk perceptions or perceived importance of social distancing behaviors. This is consistent with another study, which found that 20% of people with chronic conditions did not perceive greater risk [6], but differs from other survey reports that indicate people with different chronic conditions are more likely to engage in COVID-19 prevention behaviors and perceive COVID-19 as a serious threat [18,25]. This may be due to a close resemblance between the hypertension and general populations in our study, or it may be a result of our method of matching cases to controls rather than comparing groups without such adjustment. Another Australian survey found similarly high risk perceptions, so there may also be a ceiling effect in Australia across community groups [26].

Responses to flu vaccine uptake varied across the two groups, whereby those with hypertension were more likely to intend to vaccinate compared to healthy controls. It is possible this is due to the former’s greater exposure to the health system where doctors may mention the flu vaccine each year. This difference does not appear to transfer to increased intent for COVID-19 vaccine uptake, but this may be due to a ceiling effect with high acceptance rates in Australia [27] compared to other countries such as France [28]. It should be noted that vaccine acceptance rates are changing over time as new information (and misinformation) becomes available about the various vaccines [29] now being used around the world. No COVID-19 vaccinations were available to Australians at the time of the study in 2020.

Differences in medication use were found between groups, but this was to be expected given that preventive medication is recommended for hypertension. Surprisingly, there were no differences in access difficulties or changes to medication. The Australian Bureau of Statistics reported in April 2020 that almost half (47%) of respondents with a chronic condition had used telehealth [24], including electronic prescriptions; this was not a focus of our survey but may explain why little change was detected.

### Strengths and Limitations

The strengths of this study include a large national sample with data during and after lockdown restrictions, which enabled matched case-control analyses between participants with self-reported hypertension and healthy controls and the use of established, well-validated measures.

The sample was recruited via an online research panel and social media, and has a low proportion of culturally and linguistically diverse participants; hence, different results may be found in other populations. We are currently conducting a separate survey of these communities in their preferred language. The survey involved nonstratified sampling without targeted recruitment of specific health conditions, and only a subset were included in the longitudinal substudy. Future research could explore the influence of multimorbidity and differences between social media users and other community members, given misinformation concerns in Australia [30].

### Conclusion

Anxiety was above normal levels for all groups during the COVID-19 lockdown. This was higher among people with hypertension, who also had higher influenza vaccination intentions but similar COVID-19 vaccination intentions. In Australia, where lockdown measures effectively reduced the spread of COVID-19 and restrictions eased relatively quickly, these differences dissipated after 2 months, but locations with prolonged restrictions may require targeted psychological screening for vulnerable groups. Despite a decrease in perceived seriousness and anxiety after 2 months of lockdown restrictions, vaccination intentions for both influenza and COVID-19 remained high (80%), which is encouraging for the future prevention of COVID-19.

## Abbreviations

aOR: adjusted odds ratio
aPR: adjusted prevalence ratio
CHAI: Consumer Health Activation Index
CVD: cardiovascular disease
MMD: marginal mean difference
STAI: State-Trait Anxiety Inventory
